# Complete genome sequence of *Cupriavidus* sp. Agwp_2, isolated from soil in an iron mining area

**DOI:** 10.1128/mra.00884-24

**Published:** 2025-05-27

**Authors:** Zilin Yang, Shiping Zhang, Xiangyang Li

**Affiliations:** 1 School of Sciences, Kaili University 117805 , Kaili, China; 2 School of Biology and Environmental Engineering, Guiyang University 105797 , Guiyang, China; 3 School of Life and Health Science, Bacterial Genome Data Mining & Bioinformatic Analysis Center, Kaili University, Kaili, China; DOE Joint Genome Institute, Berkeley, California, USA

**Keywords:** *Cupriavidus* sp. Agwp_2, complete genome, silver resistance, Nanopore

## Abstract

*Cupriavidus* spp. is a Gram-negative bacterium belonging to the Burkholderia family. Here, we report the complete genome sequence of *Cupriavidus* sp. Agwp_2, whose genome consists of two chromosomes of 3,823,900 bp and 2,784,676 bp, respectively, and one plasmid of 847,469 bp with an average GC content of 66.17%.

## ANNOUNCEMENT

The genus *Cupriavidus* includes Gram-negative bacteria belonging to the Burkholderia family within the β-proteobacteria class. Strain Agwp_2 was isolated from a soil sample that was gathered from a deserted iron mining site (26.46 N 106.38 E) situated in Doupeng Mountain, Guiyang, China in July 2021. The soil sample was processed into a suspension and vigorously agitated for 2 hours. Then, 200 µL of the supernatant was plated onto a selective Luria-Bertani (LB, excluding NaCl) agar plate supplemented with 50 µmol/L AgNO3. The inoculated plate was inverted and incubated at 28°C for 48 hours. Ultimately, we obtained a colony named Agwp_2 that exhibited resistance to silver nitrate, as confirmed by its successful growth in NaCl-free LB liquid medium supplemented with 50 µmol/L AgNO_3_. The 16S rRNA gene sequence was amplified using the colony PCR method with the primer pair 27F and 1492R. A BLAST search for the 16S rRNA gene of the Agwp_2 strain showed the highest sequence identity of 99.15% to that of *Cupriavidus necator* strain N-1 (NR_102851.1); thus, it was designated as *Cupriavidus* sp. Agwp_2. This genome sequence is a valuable resource for defining the relatedness among species within the genus *Cupriavidus*.

Strain Agwp_2 was cultured in 100 mL LB medium at 28°C for 24 hours. Genomic DNA was extracted using the SDS method (1), and its quality and quantity were assessed by agarose gel electrophoresis and Qubit 2.0 Fluorometer (Thermo Fisher Scientific), respectively. The library with an insert size of ≥10 kb was prepared with the SQK-LSK109 rapid barcoding kit (Oxford Nanopore, Oxford, UK) with the native barcoding expansion kit (EXP-NBD104) following the manufacturer’s protocol. The constructed library was loaded onto a PromethION Flow Cell FLO-PRO002 (Oxford Nanopore Technologies) and sequenced on a PromethION Beta sequencer. Base calling was performed using Guppy v6.2.7 (2) with the parameter “-c dna_r9.4.1_450bps_hac_prom.cfg.” The data underwent quality control using NanoPlot (v1.38.0) (3) with the minimum Q score cutoff of 7. A total of 262,814 filtered reads (2,928,675,317 bp) were obtained, with a mean length of 11,143.50 bp and an *N*50 value of 12,155 bp, providing an average coverage of 392.79×. Sequence assembly was performed using Unicycler (Version 0.4.7) (4) with default parameters and polished using Medaka (v1.8.0) with basecall model “r941_prom_hac_g507.” The genome sequences were circularized using Unicycler that automatically identified and trimmed the overlapping ends, and the genome was not rotated. Assembly quality was assessed using both checkM v5.0.2 (5) and Quast (6) with default parameters. Taxonomic classification by average nucleotide identity analysis, using a local version of PGAP (version 2024-04-27.build7426) (7), showed a score below the cutoff value (>95%) with the closest relative (92.917%), *C. oxalaticus* NBRC 13593^T^ (GCA_001592245.1).

Two chromosomes of 3,823,900 bp and 2,784,676 bp and the plasmid of 847,469 bp are all circular, with GC content of 66.44%, 67.15%, and 61.73%, respectively. We annotated Agwp_2’s genome using the PGAP v5.3 (7). A total of 6,832 genes were identified across all three replicons, including 6,537 coding sequences, 93 RNA genes, and 202 pseudogenes.

## Data Availability

The complete genome sequence of *Cupriavidus* sp. Agwp_2 is deposited in GenBank under accession number CP088083-CP088085. Sequencing reads are available in the Sequence Read Archive (SRA) under accession number SRR29911121. The BioProject and BioSample accession numbers are PRJNA781467 and SAMN23282433, respectively.
